# Small Molecules for Enhancing the Precision and Safety of Genome Editing

**DOI:** 10.3390/molecules27196266

**Published:** 2022-09-23

**Authors:** Siyoon Shin, Seeun Jang, Donghyun Lim

**Affiliations:** 1 School of Biopharmaceutical and Medical Sciences, Sungshin University, Seoul 01133, Korea; 2 Department of Next-Generation Applied Science, Sungshin University, Seoul 01133, Korea

**Keywords:** genome editing, CRISPR, Cas nuclease, guide RNA, small molecule, specificity

## Abstract

Clustered regularly interspaced short palindromic repeats (CRISPR)-based genome-editing technologies have revolutionized biology, biotechnology, and medicine, and have spurred the development of new therapeutic modalities. However, there remain several barriers to the safe use of CRISPR technologies, such as unintended off-target DNA cleavages. Small molecules are important resources to solve these problems, given their facile delivery and fast action to enable temporal control of the CRISPR systems. Here, we provide a comprehensive overview of small molecules that can precisely modulate CRISPR-associated (Cas) nucleases and guide RNAs (gRNAs). We also discuss the small-molecule control of emerging genome editors (e.g., base editors) and anti-CRISPR proteins. These molecules could be used for the precise investigation of biological systems and the development of safer therapeutic modalities.

## 1. Introduction

Modern genome-editing tools, particularly CRISPR-Cas technologies, have made transformative changes in biology, biotechnology, and medicine. Researchers can alter the genome of live cells to generate designer cells for studying biological phenomena and developing new therapeutics [1,2].

**Figure 1:**
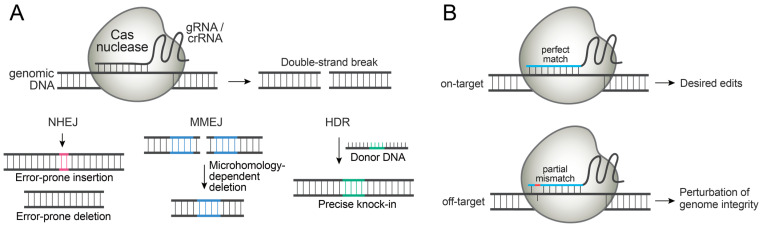
(**A**) Cas-nuclease-induced double-strand break (DSB) is repaired by endogenous cellular pathways. (**B**) Cas nucleases often tolerate partial mismatches and induce off-target DNA cleavages.

Cas nucleases generate a double-strand break (DSB) at the specific genomic locus directed by gRNAs or CRISPR RNAs (crRNAs). Once the genomic DNA is cleaved, endogenous DNA repair pathways are immediately activated to ligate the broken DNA ends (Figure 1A). The most efficient pathway, non-homologous end joining (NHEJ), introduces small insertions or deletions to rejoin the broken ends. The microhomology-mediated end joining (MMEJ) pathway can introduce small deletions by recognizing the homology on either side of the DSB. When a donor DNA comprised of homology arms and desired edits is introduced along with Cas nucleases, the templated DNA repair pathway, homology-directed repair (HDR), can introduce the desired edit. NHEJ and MMEJ can be exploited for gene knock-out, and HDR can be exploited for introducing precise knock-in, though the efficiency of HDR is generally low [3,4,5].

To solve the problem of low HDR efficiency, base editors and prime editors have been developed for placing desired edits without DSB [6,7]. They are versatile tools for small-sized base replacements, insertions, and deletions. However, they are not applicable to inserting large DNA fragments, and their activities are not robust across diverse cell lines or genomic loci. Thus, HDR should be used in many genome-editing scenarios, necessitating the precise control of Cas nucleases to minimize side effects arising from unintended DSB.

Regardless of the type of desired edits, a key to successful genome editing is to exclusively place the edits at specific genomic loci. However, the prolonged activity of Cas nucleases can lead to off-target DNA cleavage and permanently alter unintended genomic loci, sometimes with detrimental consequences (Figure 1B). Off-target DNA cleavage can also lead to chromosome rearrangements and genotoxicity [8,9]. In addition, continued Cas nuclease expression may induce immune responses in humans due to the pre-existing adaptive immunity to Cas9 proteins [10,11]. Moreover, Cas9 expression could interfere with host protein expression [12].

Various methods have been developed to increase the intrinsic specificities of the genome-editing machinery by engineering Cas nucleases or gRNAs. For example, diverse SpCas9 variants with enhanced specificity have been reported [13,14]. Chemical modification of gRNAs or engineering the gRNA secondary structure can improve the on-target specificities [15,16,17]. However, these strategies may diminish the Cas9 activities, and the off-target effect cannot be completely abolished.

Only a few copies of Cas nuclease substrate are present in cells, and the nucleases display faster kinetics at on-target sites than at off-target sites [18,19]. Thus, precise and safe genome editing can also be achieved by maintaining the activity or concentration of genome-editing machinery in a narrow temporal window. For example, anti-CRISPR (Acr) proteins can be applied to switch off genome editing once the desired edits are placed, thereby minimizing off-target DNA cleavages. However, the use of Acr proteins is restricted since they cannot cross the cell membranes [20,21].

Small molecules are powerful tools for modulating biological phenomena as they are cell-permeable to enable precise temporal controls [22]. Moreover, their action is readily reversible for sequentially turning on and turning off the target biomolecules. They are easy to implement, inexpensive, and non-immunogenic. Not surprisingly, small molecules have been preferred agents for drug development. Genome-editing technologies, like other biological systems, could greatly benefit from small molecules. For example, small-molecule Cas9 degraders can remove Cas9 from the cells once its job is done.

Here we provide a comprehensive overview of small molecules for controlling genome-editing systems. Basic ideas for small-molecule control of Cas9 have been reviewed elsewhere [23,24]. Therefore, this review discusses recent applications of the small-molecule control systems as well as the principles of each system. In addition, recent additions to the repertoire of small-molecule controllers are presented. Particularly, we cover Cas9 as well as emerging genome-editing tools (i.e., Cas12, base editors, prime editors, dCas9-based epigenetic modifiers, and anti-CRPSR proteins) controlled by small molecules. We also discuss how the small molecules could enhance the genome-editing specificity.

The presented control systems could readily be applied in each laboratory since many of the small molecules are commercially available, and Cas nuclease and gRNA constructs are publicly shared or easy to generate. From this review, readers will become familiar with diverse small-molecule tools for controlling Cas nuclease and gRNA and choose appropriate tools for their genome-editing scenario.

## 2. Small-Molecule Control of Cas Nuclease and gRNA Expression Levels

Various systems for small-molecule modulation of transcript levels have been developed long before the advent of CRISPR technology. For example, small molecules can modulate the activity of transcription factors, thereby amplifying or suppressing the transcription. Methods for controlling the translation of the target transcript have also been extensively studied. These tools can directly be applied to controlling the production of Cas nucleases or gRNAs. Here, we provide an overview of recent examples modulating the expression of genome-editing machinery.

### 2.1. Small-Molecule Control of Cas Nuclease Expression Levels

**Figure 2:**
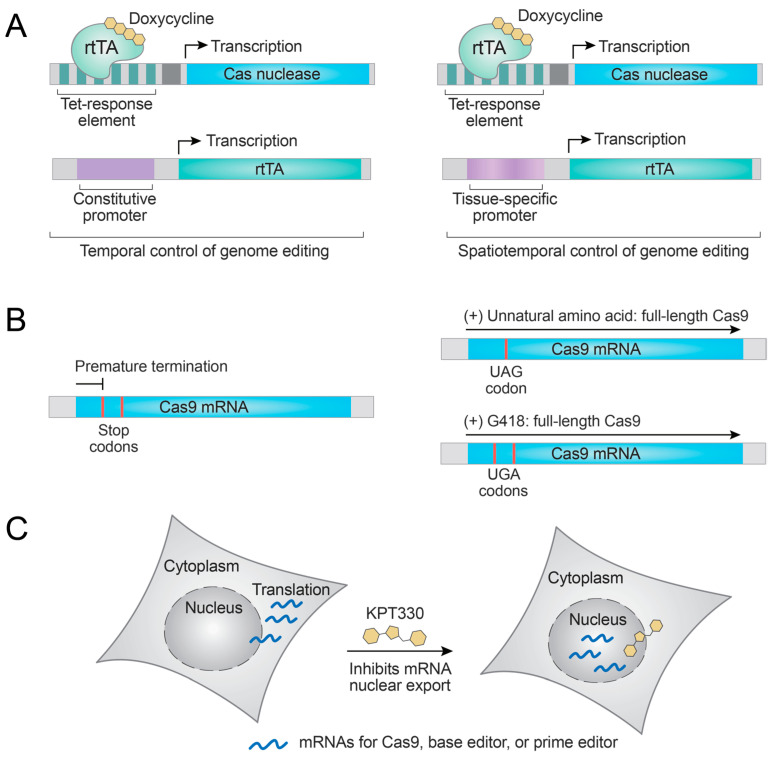
Examples of the small-molecule control of Cas nuclease expression. (**A**) Transcriptional control of Cas nuclease expression by doxycycline. Temporal or spatiotemporal control of the nuclease expression can be achieved by employing appropriate promoters for rtTA expression. (**B**) Small-molecule control of Cas9 translation using stop-codon read-through strategies. (**C**) Small-molecule control of mRNA export to modulate the expression of genome-editing machinery.

The doxycycline-inducible gene expression or repression system is the most widely used method for controlling the transcript level. An early example employed this system to regulate the synthesis of SpCas9 mRNAs, thus SpCas9 concentration and gene modification frequencies in vitro and in vivo (Figure 2A) [25]. Cao et al. used the doxycycline-inducible system to narrow down the SpCas9 expression within a specific time window and demonstrated that off-target editing was significantly reduced while maintaining on-target editing efficiency [26]. One of the advantages of the doxycycline-inducible system is that various cell lines stably integrated with doxycycline-inducible SpCas9 are available, such that functional studies by gene knock-out or CRISPR screens can readily be performed. Example studies include cancer drug discovery, investigating ubiquitin ligases in mitosis, and identifying transcriptional regulators of telomerase, to list a few [27,28,29]. In addition, tissue-specific rtTA delivery or tissue-specific promoter-driven rtTA expression can be used for tissue-specific SpCas9 expression, thus achieving spatiotemporal control of genome editing (Figure 2A) [25,30]. Similar to SpCas9, other genome editors such as SaCas9, Cas12a, base editor, and prime editor can also be expressed under the control of doxycycline [31,32,33,34]. Another advantage of these systems is that doxycycline is an FDA-approved antibiotic. Thus, its effects on humans are well understood to allow in vivo therapeutic genome editing.

Even though the doxycycline-inducible system is highly reliable and widely used, it has several disadvantages. The requirement for an extra rtTA expression cassette complicates the system, and the turn-on kinetics are slow since both transcription and translation are required. For faster induction by small molecules, Cas nuclease expression can be modulated at the translational level. Suzuki et al. coupled Cas9 translation with an unnatural amino acid incorporation system [35]. When the SpCas9 mRNA is intervened by an amber codon (UAG), the full-length SpCas9 can be produced only when the cells are supplied with the unnatural amino acid and the amber codon is suppressed. They proved this idea by developing a Lys(Boc)-dependent genome-editing system in mouse preimplantation embryos (Figure 2B) [35]. Even though this system allows faster induction of SpCas9 production, the requirement for an orthogonal aminoacyl-tRNA synthetase (aaRS)/tRNA pair complicates the system. Yaméogo et al. designed the ”CRISPR-Stop Codon Read Through” system wherein the Cas9 expression cassette contains two stop codons (UGA) in the reading frame. Here, aminoglycoside antibiotics such as G418 induced the read-through of the two stop codons and initiated the production of full-length SpCas9 or CjCas9, thereby achieving the correction of *DMD* and *FXN* genes (Figure 2B) [36]. Although the system is minimal without any extra factors, in vivo nephrotoxicity of G418 may hamper its practical applications [37].

Cas9 mRNAs should be exported from the nucleus to the cytoplasm for translation. Cui et al. performed a cell-based screening of a chemical library to identify SpCas9 inhibitors and found that an FDA-approved drug KPT330 and its analogs inhibit SpCas9-mediated genome editing in human cells [38]. Mode-of-action studies revealed that KPT330 inhibits the nuclear export of Cas9 mRNA and blocks its translation, in accordance with its well-known mechanism. Interestingly, KPT330 could enhance the on-target specificity of SpCas9-based genome editing, and enhanced the specificity of cytosine base editors by displaying the inhibitory effects on the out-of-window cytosines more than on the on-target cytosine, although these enhancements were modest. KPT330 also blocked the gene modification by prime editors (Figure 2C) [38]. KPT330, however, was originally developed as an exportin-1 inhibitor and may affect the global transport of endogenous mRNAs and other type of RNAs [38]. These effects should be investigated in more detail for their practical applications. In addition, these translational control methods still display slower dynamics compared to methods that target the existing Cas9 proteins.

### 2.2. Small-Molecule Control of gRNA Expression Levels

Similar to the transcriptional modulation of Cas9 expression, gRNA expression can be modulated by doxycycline-inducible systems. For example, doxycycline-inducible gRNA expression cassette under H1/TetO promoter allowed the temporal control of neuronal genome editing and the identification of tumor-promoting mutations in vivo [39,40]. In these systems, the administration of doxycycline blocked the binding of Tet repressor (TetR) to the H1/TetO promoter, thus initiating gRNA transcription and SpCas9-mediated genome editing. Similarly, SaCas9 activity can be switched on by doxycycline-inducible gRNA expression for in vivo genome editing in neurons [41]. Although the doxycycline-inducible gRNA expression system is robust across diverse cell types, leaky expression of the target transcript is sometimes observed [39]. Moreover, Cas9 protein should be constitutively expressed, which may promote the immune response to the foreign protein.

Kelkar et al. reported a self-inactivating CRISPR system wherein a gRNA targeting the SpCas9 expression cassette was produced in a doxycycline-dependent manner [42]. Thus, doxycycline switched off genome editing by blocking SpCas9 expression. This strategy enhanced the editing specificity by suppressing the off-target cleavage more than the on-target cleavage [42]. However, this system requires the expression of an extra gRNA, and intracellular Cas9 protein levels are diminished slowly requiring several days of doxycycline treatment [42]. As a result, a strict temporal control of Cas9 protein may not be available.

In another study, inducible activation of Cre-ERT2 recombinase by 4-hydroxytamoxifen (4HT) resulted in the recombination of gRNA expression cassettes harboring loxP sites. This recombination could promote or block the synthesis of functional gRNAs [43]. Particularly, 4HT-induced suppression of the genome editing two days after the delivery of editing machinery significantly reduced the off-target cleavages, while the on-target cleavage efficiency was unaffected or minimally affected [43]. This study demonstrated the importance of disrupting the genome editing at a specific time point for precise and safe editing. However, the use of this system may be restricted due to the requirement for an extra expression of the recombinase.

## 3. Small-Molecule Control of Cas Nucleases

Transcriptional and translational controls are generally slower than controlling biomolecules already present in cells because transcription, translation, and nuclear translocation are required to initiate genome editing. In addition, extra factors such as rtTA, TetR, unnatural amino acid incorporation machinery, or recombinase may be required. Doxycycline-controlled systems could display leaky expression of the target [39]. Finally, existing nucleases and gRNAs cannot be removed immediately even when the switch-on signal is removed or the switch-off signal is added. Thus, a great deal of effort has been put into designing post-translational control systems for Cas nucleases, such that rapid, temporal, and reversible control of genome editing is realized.

Native Cas nucleases can be targeted by small molecules, as exemplified by small-molecule SpCas9 inhibitors [21]. On the other hand, engineered Cas nucleases containing a handle for small-molecule modulation can be employed for precision control. This section describes such efforts by introducing basic ideas and the most recent examples.

### 3.1. Small-Molecule SpCas9 Inhibitors

**Figure 3:**
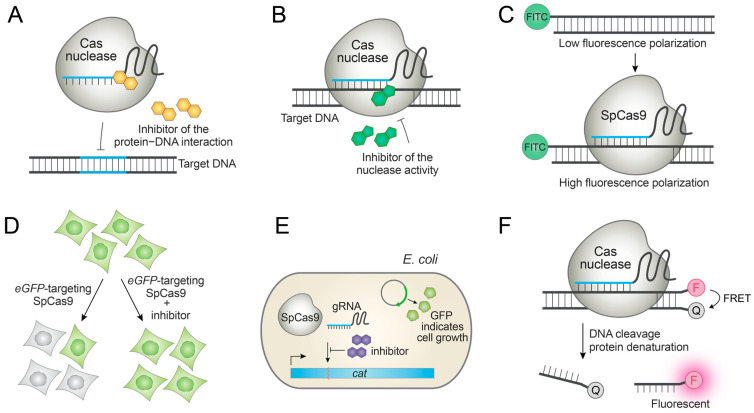
Discovery of anti-CRISPR molecules to inhibit native Cas nucleases. (**A**,**B**) Anti-CRISPR molecules display different modes of action, including (**A**) inhibition of the Cas nuclease−DNA interactions, and (**B**) inhibition of the nuclease domains. (**C**) A high-throughput in vitro fluorescence polarization assay to detect SpCas9−PAM (protospacer adjacent motif) interactions. (**D**) An image-based high-content assay to identify SpCas9 inhibitors in human cells. (**E**) A cell-based assay to identify SpCas9 inhibitors in *E. coli*. (**F**) A high-throughput in vitro FRET assay to identify SpCas9 inhibitors.

CRISPR is a bacterial immune system to fight against invading phages. In response to the bacterial CRISPR systems, phages have evolved anti-CRISPR (Acr) proteins to disable the CRISPR systems (Figure 3A,B) [20,44]. Acr proteins have been extensively studied and repurposed as tools to switch off Cas nucleases for safer genome editing. However, the proteinous nature of the current Acrs hampers their timely delivery into cells. As a result, researchers have been developing high-throughput screening platforms to identify synthetic Acr small molecules.

Maji et al. developed a fluorescence polarization (FP)-based high-throughput assay to identify small molecules that inhibit the interaction between SpCas9 and its NGG protospacer adjacent motif (PAM) sequence (Figure 3C) [21]. For investigating the cellular activity of the hit compounds, they also optimized an image-based high-content assay in an eGFP-expressing stable cell line wherein the *eGFP*-targeting SpCas9 can be inhibited by small molecules. Thus, the active inhibitors give rise to a high eGFP signal, while inactive compounds give a low eGFP signal (Figure 3D). Using these assays, they identified a series of small-molecule inhibitors of the SpCas9−PAM interaction after extensive structure–activity relationship (SAR) studies of the initial hit [21]. In accordance with their mode of action, the compounds inhibited SpCas9-mediated DNA cleavage, base editing, and dSpCas9-based transcriptional activation [21]. This is the first report of small-molecule SpCas9 inhibitors having cellular activities, and demonstrates the possibility of controlling SpCas9 with synthetic Acr small molecules. However, the efficacy and potency of the compound is sub-optimal, requiring the identification of new chemical scaffolds.

Lee et al. reported a cell-based high-throughput screening platform in *E. coli*, wherein *cat* locus conferring chloramphenicol resistance is disrupted by SpCas9 and small-molecule SpCas9 inhibitors can rescue chloramphenicol-mediated cell death by protecting the *cat* gene from the cleavage (Figure 3E). The hit compound was subjected to SAR studies to give several small molecules that are functional in *E. coli* [45], although their activity in eukaryotic cells has yet to be determined. These inhibitors were proposed to bind apo-Cas9 and prevent gRNA loading [45].

Seamon et al. reported an in vitro Förster resonance energy transfer (FRET) assay wherein each strand of the dsDNA substrate is labeled with a fluorophore and a quencher [46]. Because SpCas9, SaCas9, and CjCas9 are still bound to the substrate even after the dsDNA cleavage, a protein denaturant such as guanidine hydrochloride is added to the reaction mixture to denature Cas9. This leads to the release of the cleaved products that spontaneously dissociate into ssDNAs. Consequently, the fluorophore-labeled and quencher-labeled oligonucleotides are dissociated from each other, giving a high fluorescence signal (Figure 3F). High-throughput screening of chemical libraries using the assay gave several hit compounds that inhibit SpCas9 in vitro, though their cellular activities could not be measured due to the high cytotoxicity [46].

Valproic acid was identified as a SpCas9 binder from a screening campaign using a protein thermal shift assay [47]. The compound bound to SpCas9 and decreased its melting temperature. Therefore, valproic acid-induced SpCas9 denaturation was observed particularly at higher temperatures. Cellular activity of the compound was demonstrated. However, it required extra measures (e.g., photothermal triggers such as indocyanine green irradiated with a near-infrared laser) for efficiently removing the protein [47].

In addition to acting as a safety measure to control genome editing, Acr molecules could be used as antibiotics against drug-resistant bacteria [48,49]. Thus, many more small-molecule inhibitors against Cas nucleases are expected in the near future, with the concomitant development of high-through assays suitable for each nuclease [50].

### 3.2. Targeted Degradation of Cas Nucleases by Small Molecules

Degradation of a target protein using bifunctional small molecules or molecular glues is emerging as the next-generation modality in small-molecule therapeutics [51]. The basic ideas of targeted degradation have been successfully demonstrated for Cas nucleases. Kleinjan et al. employed an auxin-inducible degron (AID) system for dCas9 degradation [52]. They fused dCas9-based transactivators to AID and modulated the level of transcriptional activation using auxin. Strikingly, auxin caused rapid degradation (~30 min) of the targets to enable timely regulation of the Cas proteins. In addition, dose-dependent precise control was demonstrated, which is the key advantage of small-molecule control [52]. However, AID is a plant-derived system requiring the extra expression of plant factors for ubiquitination when the system is used in mammalian cells.

**Figure 4:**
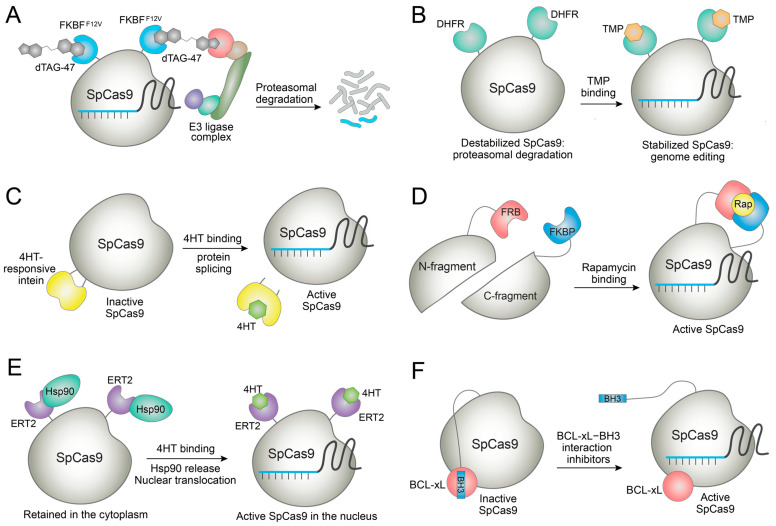
Examples of small-molecule control of engineered Cas nucleases. (**A**) Targeted degradation of SpCas9-FKBP^F12V^ fusion by dTAG-47 molecule to switch off genome editing. (**B**) Destabilized SpCas9-DHFR fusion is stabilized by TMP to switch on genome editing. (**C**) SpCas9 fused to an engineered intein is spliced by binding to 4HT, and the active SpCas9 is released. (**D**) Split SpCas9 is dimerized by rapamycin to reconstitute active SpCas9. (**E**) SpCas9-ERT2 fusion in the cytoplasm is translocated to the nucleus upon binding of 4HT to the ERT domain. (**F**) SpCas9 activity is blocked by the autoinhibitory BCL-xL−BH3 interaction but restored by the inhibitors of the protein−peptide interaction.

Proteolysis-targeting chimera (PROTAC) is a representative example of bifunctional small molecules for targeted degradation. Using the principles of PROTAC, dTAG molecules have been developed for the specific degradation of FKBP^F36V^-containing proteins in cells [53,54]. Sreekanth et al. employed SpCas9-FKBP^F36V^ fusion for genome editing and used the dTAG-47 molecule for degrading the fusion protein (Figure 4A) [55]. Owing to the fast kinetics of the dTAG system, SpCas9 activity could be terminated nearly by 90% in different cellular assays. The SpCas9 degradation enhanced the on-target specificity of the editing. Interestingly, SpCas9 degradation affected genome editing outcomes, namely the choice of NHEJ, MMEJ, or HDR [55]. SpCas9-FKBP^F36V^ was also delivered as ribonucleoprotein (RNP) that generally exhibits a lower off-target effect [56]. Thus, further increase in the editing specificity would be possible. This system does not require extra factors because it uses the endogenous ubiquitin–proteasome pathway in human cells. The FKBP^F36V^ tag may be fused to any Cas nucleases such that this strategy could find widespread use for rapid deactivation of various CRISPR systems and for directing DNA repair in a specific way. However, dTAG molecules could induce the degradation of endogenous zinc finger proteins [54], requiring the careful investigation of dTAG’s effects on cellular fitness.

Gama-Brambila et al. engineered several Cas proteins (SpCas9, dSpCas9, PdCas12a, and LwCas13a) to contain a Phe-Cys-Pro-Phe (FCPF) sequence. When cells are treated with a lenalidomide-conjugated perfluoroaromatic compound, named PROTAC-FCPF, the cysteine residue of the FCPF moiety is connected to the compound and the Cas proteins are degraded by the proteasomal pathway [57]. Like the dTAG system, the possibility of degrading endogenous zinc finger proteins should be carefully investigated when applying this system. That being said, we anticipate that these and emerging protein degradation strategies could be practically employed for rapid control of Cas9′s half-lives, given the unprecedented speed of PROTAC discovery.

### 3.3. Conditional Stabilization of Cas Nucleases by Small Molecules

As opposed to the induced degradation system, Cas nucleases can be conditionally stabilized by small molecules. For example, destabilizing domains are fused with Cas nucleases such that the fusion proteins are rapidly degraded to minimize background nuclease activities. Genome editing is initiated when small-molecule stabilizers bind to and stabilize the fusion domain. This system enables fast upregulation of genome editing in cells. Maji et al. fused the *E. coli* dihydrofolate reductase (DHFR) domain or estrogen receptor (ER50) domain to SpCas9 [58]. These fusions are rapidly cleared by the ubiquitin–proteasome pathway. When the cells are treated with trimethoprim (TMP) that binds to the DHFR domain or 4-hydroxytamoxifen (4HT) that binds to the ER50 domain, the fusions become stabilized and protected from degradation, and SpCas9 becomes active (Figure 4B). This system enables substantial on-target DNA cleavages while greatly suppressing off-target cleavages [58]. In practice, the Cas9-DHFR system was employed to control gene-drive inheritance to solve the safety issue of the technology [59]. Yan et al. generated nanoparticles containing SpCas9-DHFR plasmids and encapsulated the nanoparticles in a macrophage-derived membrane [60].The membrane was embedded with a prodrug of TMP that releases active TMP upon stimulation by reactive oxygen species. Thus, this system allows SpCas9 activation under an inflammatory environment to enable in vivo colon-specific genome editing for the treatment of inflammatory bowel disease [60]. Manna et al. demonstrated spatiotemporal control with the SpCas9-DHFR construct using a caged TMP that is uncaged by visible light [61]. The Cas9-DHFR system displayed background genome editing in the absence of the ligand, or maximum Cas9 activity could not be attained in some cases [58]. That being said, its usefulness was demonstrated in various settings, and the effects of TMP on humans are well understood since it is an FDA-approved antibiotic. Thus, we expect that Cas9-DHFR fusion would find widespread use in therapeutic genome editing.

Similarly, SpCas9 is fused to a destabilizing domain (DD), a mutant derived from the FKBP12 protein. The resulting DD-SpCas9 is rapidly degraded by the proteasomal pathway. A small molecule Shield-1 binds to the DD and protects the fusion protein from degradation. This way, SpCas9 activity could be induced by Shield-1 in vitro and in vivo [62,63]. Moreover, the DD is fused to dSpCas9-VPR to enable reversible control of gene expression [64]. This system displayed minimal or no genome editing in the absence of Shield-1 [62], although its effect on humans should be investigated in more detail for therapeutic applications.

### 3.4. Small Molecule-Mediated Release of Functional Cas Nucleases

Ligand-dependent intein systems can be used for controlling genome editing [65]. Here, SpCas9 was fused to an evolved intein that is responsive to 4-hydroxytamoxifen (4HT). The intein fusion site on SpCas9 was rationally chosen to block the SpCas9 activity by the fusion. When 4HT is added, the protein splicing takes place and active SpCas9 is released (Figure 4C). Using this system significantly improved the genome-editing specificity by greatly reducing off-target DNA cleavages, though a reduction in the on-target editing was sometimes observed [65]. 4HT is a clinically used estrogen receptor modulator with known side effects [66]. Thus, care should be taken for therapeutic application of this and other 4HT-inducible genome-editing systems.

Several groups reported systems wherein SpCas9 was split into two fragments (N-Cas9 and C-Cas9), and each fragment was fused with FRB or FKBP. Thus, functional SpCas9 is assembled by rapamycin, which dimerizes FRB and FKBP [67,68,69]. A similar strategy was applied for the rapamycin control of Cas12a (Figure 4D) [70]. Split base editors could be generated by splitting the deaminase domains instead of the Cas9 domain for minimizing the genome-wide off-target effect arising from the constitutively active, promiscuous deaminase activity [71,72]. These systems displayed low background editing to enable tight control of genome editing, although care should be taken in the use of rapamycin since it is an immunosuppressant.

Nguyen et al. added an extra layer of control for minimizing the background editing of the rapamycin-inducible system [68]. When each split fragment is further fused to a ligand-binding domain from estrogen receptor (ERT), the fragments are sequestered in the cytoplasm due to the binding between ERT and cytosolic Hsp90. When 4HT binds to ERT, Hsp90 is displaced and the split fragments are localized to the nucleus to assemble with gRNA to form a functional RNP complex. This dual small-molecule control system using rapamycin and 4HT displayed minimal background activity to enable tight control of genome editing as well as dSpCas9-VPR-based transcriptional activation [68].

Similarly, SpCas9-ERT2 and AsCas12a-ERT2 fusions are sequestered in the cytoplasm but localized to the nucleus by binding to 4HT (Figure 4E) [73,74]. Importantly, controlling the duration of 4HT treatment could enhance the specificity of the SpCas9-mediated editing because off-target cleavage is emerging later than on-target cleavage [73,74]. However, the background activity of the 4HT-mediated nuclear translocation system should be further reduced for tighter control of genome editing.

Rose et al. designed a chemically inducible Cas9 (ciCas9) system rapidly activated within minutes [75]. Here, SpCas9′s nonessential REC2 domain was replaced by BCL-xL, and the BH3 peptide was fused to the C-terminal of SpCas9 to introduce autoinhibitory BCL-xL–BH3 interaction. Small molecule inhibitors of the BCL-xL–BH3 interaction such as A-385358, A-1155463, and WHEI-539, blocked the autoinhibitory interaction and activated the SpCas9 (Figure 4F) [75]. The genome editing was induced with fast kinetics since only the disruption of the protein–peptide interaction was required. An increase in the target specificity was demonstrated with this system even though a reduction in the on-target editing was also observed. Interestingly, applying this strategy to a SpCas9 variant carrying specificity-enhancing mutations completely abolished the off-target editing at the *EMX1* locus [75,76]. This idea was expanded for small-molecule control of transcriptional activators, base editors, and prime editors [77]. Unlike the other Cas9 fusion proteins, ciCas9 is generated by the domain replacement. Thus, its size is similar to wild-type Cas9 [77], conferring an advantage in viral-vector-mediated delivery. However, the BCL-xL–BH3 interaction inhibitors could display toxicity [78]. Therefore, their effects on the target cells or organisms should be investigated before using the ciCas9 systems.

Luo et al. employed an unnatural amino acid incorporation system to replace K866, which is important for SpCas9 catalysis with an ortho-azidobenzyloxycarbonyl lysine (OABK), a protected form of lysine. Staudinger reduction mediated by 2-(diphenylphosphino)benzoic acid (2DPBA) or 2-(diphenylphosphino)benzamide (2DPBM) removes the protecting group of OABK and restores the native K866 to restore SpCas9 activity [79]. In a similar strategy, a cytosine base editor was masked by replacing K1200 with a bulky trans-cyclooctene-caged lysine (TCOK) to block the nCas9–PAM interaction. When TCOK is bioorthogonally reacted with 1,4-dimethyl-2,3,5,6-tetrazine (Me_2_Tz), the functional lysine is restored and base editing is initiated [80]. Even though these systems require an extra expression of unnatural amino acid incorporation machinery (i.e., orthogonal aaRS and tRNA) in the case of DNA delivery, RNP-based genome editing could be employed for simplifying the system.

## 4. Small-Molecule Control of gRNAs

For in vivo genome editing, Cas9 and gRNA expression cassettes are packaged into an adeno-associated virus (AAV) vector. However, AAV has a limited packing capacity of ~4.7 kDa [81]. Thus, many genome editing cassettes (e.g., genes for SpCas9 and gRNA, and their regulatory elements) are packaged into dual AAV vectors. For safer and more efficient delivery, the genome-editing machinery needs to be packed in a single AAV vector. Therefore, much effort is being put into identifying and engineering CRISPR systems with smaller sizes [82].

Fusing a protein domain to Cas9 for small-molecule control can significantly increase the transgene size and hamper efficient viral packaging. Considering this, gRNA engineering could be a viable alternative to achieve small-molecule control of genome editing. For example, small-molecule-binding aptamers can be fused with gRNA, and the aptamer can serve as a handle for controlling the overall Cas9 activity. Since aptamer-coding genes are much shorter than protein-coding genes, gRNA engineering could solve the problem of the large transgene. gRNA engineering could also solve the problem of the slow kinetics of transcriptional control because small molecules directly bind to preformed gRNAs and immediately change their functions. In this section, we describe recent progress in genome-editing control using small-molecule-responsive gRNAs.

### 4.1. Small-Molecule Control of Aptamer-Fused gRNAs

The aptamer is a small single-stranded DNA or RNA that specifically binds to its cognate target molecules such as proteins, nucleic acids, carbohydrates, or small molecules. Generally, gRNAs are comprised of a DNA-binding spacer, stem, and loop regions. These loops can be engineered to contain extra aptamer RNA sequences since many loops are solvent-exposed and tolerant of several mutations [83]. A prominent example is the dCas9-based transcriptional activation system wherein gRNAs fused with MS2-binding aptamer are employed. When an MS2-transcriptional activation domain fusion protein (e.g., MS2-VP64) is expressed in the cell along with dCas9 and the aptamer-modified gRNA, the fusion protein binds to the aptamer and induces the transcription at the gRNA-targeted locus [84].

**Figure 5:**
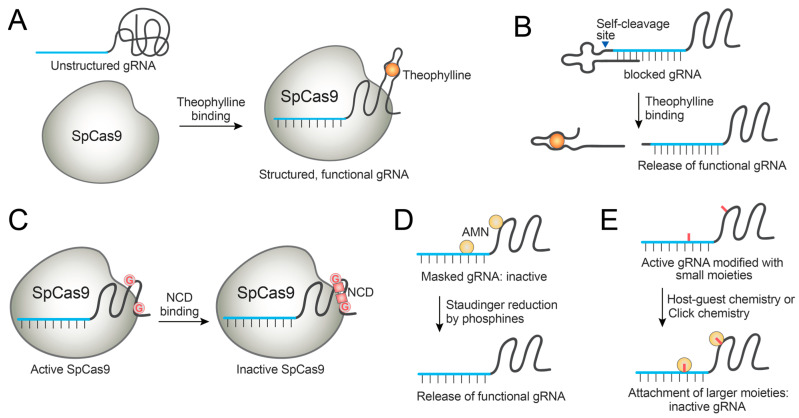
Examples of small-molecule control of engineered gRNAs. (**A**) gRNAs are fused with an aptamer that is unstructured, but are stabilized and folded into a functional form by binding to theophylline. (**B**) gRNAs are fused with an aptazyme that is activated by binding to theophylline and induces the RNA self-cleavage to release functional gRNAs. (**C**) Mutant gRNAs containing G-G mismatches are recognized and inactivated by NCD. (**D**) A gRNA masked with AMN groups becomes activated by reacting with phosphines. (**E**) Active gRNAs that contain small chemical modifications become inactive by the binding or reaction with larger moieties.

In a similar strategy, small molecule-binding aptamers were fused to gRNAs as handles for modulation. For example, theophylline-binding aptamer was fused to the solvent-exposed gRNA loops [83]. Theophylline was chosen as the small-molecule modulator since it is an FDA-approved drug not endogenously produced, and its binding to the aptamer is well-characterized [83]. By systematically introducing the aptamers to the different gRNA loop regions, several engineered gRNAs were identified that become activated or deactivated by theophylline binding (Figure 5A). Theophylline-mediated Cas9 control was demonstrated in in vitro DNA cleavage assays and in a dCas9-based repression system in *E. coli* [83]. Iwasaki et al. employed a similar design to develop another theophylline-mediated Cas9 regulation system for temporal control of genome editing in *E. coli*, in an effort to reduce editing-triggered cell death currently hampering bacterial genome editing [85]. Liu et al. also demonstrated theophylline-mediated gRNA activation using another RNA-aptamer fusion and investigated a dCas9-based gene expression system stimulated by theophylline in HEK293T cells [86]. Even though this strategy was demonstrated in a human cell line, the dynamic range of the modulation is narrow. Thus, further optimization is needed for the practical application of the system in mammalian cells.

Lin et al. developed another design for gRNA-aptamer fusion, wherein the gRNA is extended at its 3′ end with a gRNA-blocking motif and a theophylline aptamer [87]. The gRNA-blocking motif induces the generation of non-functional gRNA conformation. When theophylline binds to the aptamer, the overall gRNA conformation is altered to release the gRNA-blocking motif and generate active gRNA. The performance of this system was demonstrated in HEK293T cells, though further optimization is required for its practical application [87].

Tang et al. developed a gRNA appended with a ligand-responsive self-cleaving catalytic RNA (aptazyme) at the 5′ end [88]. When bound to theophylline, this fusion RNA displays proper conformation such that RNA self-cleavage occurs to generate a functional gRNA. Theophylline-induced activation of SpCas9 and a cytosine base editor (BE3) was demonstrated in cells, though the maximal editing efficiency was not achieved when compared with an unmodified system [88]. Similarly, guanine-binding aptamer was used to generate the aptazyme-embedded gRNA, and the guanine responsiveness was demonstrated for dCas9-VPR-mediated gene activation (Figure 5B) [88].

### 4.2. Small-Molecule Control of gRNA Mutants

Instead of fusing an aptamer, gRNA can be modified by point mutations at the stem-loop backbone, and these mutated bases can serve as a handle for small-molecule control. Liu et al. reported a gRNA-engineering strategy wherein C-to-G point mutations were introduced into the stem-loop region of gRNA, thus introducing G-G mismatches [89]. A mismatch-binding ligand, naphthyridine carbamate dimer (NCD), binds to the rationally introduced mismatches by hydrogen bonding and renders the gRNA inactive. Thus, genome editing can be switched off by NCD, which was demonstrated in HeLa cells at micromolar concentrations of NCD (Figure 5C) [89]. In the future direction, mismatch-binding ligands with improved selectivity should be identified, since NCD binds to guanines in both DNA and RNA and displays toxic side effects [89].

### 4.3. Small-Molecule Control of Chemically Modified gRNAs

Synthetic organic chemistry allows the facile generation of chemically modified gRNAs. These modifications can be used as handles for small-molecule control. Wang et al. designed chemically masked gRNAs by attaching azidomethylnicotinyl (AMN) groups to block the gRNA function [90]. When the AMN group is released by Staudinger reduction with 2-(diphenylphosphanyl)benzamide (2DPBM), the functional gRNA is released and genome editing takes place (Figure 5D). 2DPBM-mediated genome editing was demonstrated in human cells. Moreover, the crRNA of Cas13a could be protected and deprotected using the same chemistry, proving the possibility of small-molecule control of gRNAs and crRNAs from diverse CRISPR systems [90]. Habibian et al. employed the same kind of gRNA blocking, with the deprotection by TPPMS or THPP to restore functional gRNAs and genome editing in human cells [91]. Recently, a similar strategy was applied for developing conditional LbCas12a-based sensors to detect Mn^2+^ in live cells [92].

Xiong et al. reported an adamantoylated gRNA that can be deactivated by host–guest complexation with CB7 [93]. gRNAs show tolerance to adamantoylation, but host–guest complexation with CB7 leads to the gRNA modification with bulky groups that inactivate gRNAs. This strategy was demonstrated in human cells to switch off genome editing. Moreover, the system was expandable to the crRNAs of Cas13a (Figure 5E) [93].

The same research group reported clickable gRNAs that are chemically labeled to contain azido groups [94]. The labeled gRNAs are still active due to the small-sized chemical modification. These gRNAs can be inactivated by reacting with a bulky dibenzocyclooctyne (DBCO)-containing small molecule. Molecules having two or three DBCO groups induced crosslinking between gRNAs and displayed higher potency than those having a single DBCO. This strategy was also demonstrated in human cells to inhibit genome editing, and it was expandable to the crRNA of Cas13a (Figure 5E) [94].

Since chemically modified RNAs are employed, above systems would not be applied to in vivo genome editing where AAV-mediated gene delivery is employed. In addition, high concentrations of the ligands were required to switch on or switch off the genome editing, which increases the chances of toxic side effects. That being said, identification and optimization of new gRNA–ligand pairs would enable facile control of RNP-based ex vivo genome editing.

## 5. Small-Molecule Control of Anti-CRISPR Proteins

Anti-CRISPR (Acr) proteins are highly potent inhibitors of Cas nucleases. However, timely delivery of Acr proteins into cells is challenging. To solve this problem, Acr proteins can be engineered to be small molecule-responsive and delivered into cells along with Cas nucleases. In this way, small molecules can activate or deactivate Acr proteins, thus deactivating or activating Cas nucleases. Since Acr proteins are much smaller than Cas nucleases (e.g., the size of SpCas9 is ~160 kDa while the size of AcrIIA4 is ~10 kDa) [95], small-molecule control of engineered Acr proteins may be more efficient than controlling engineered Cas nucleases. Moreover, the enormous diversity and high potency of Acr proteins could allow their widespread use for therapeutic genome editing.

### 5.1. Conditional Activation of Acr Proteins by Small Molecules

**Figure 6:**
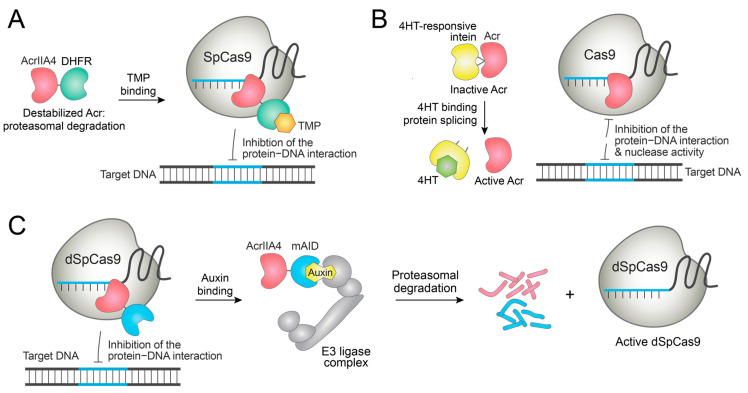
Examples of the small-molecule control of engineered Acr proteins. (**A**) Destabilized AcrIIA4-DHFR fusion is stabilized by TMP to switch off genome editing. (**B**) Acr proteins fused to an engineered intein are spliced by binding to 4HT, and the activated Acr proteins inhibit genome editing. (**C**) AcrIIA4 fused to mAID is degraded by auxin that acts as a molecular glue in plant cells, and dSpCas9 is activated.

Jain et al. generated an AcrIIA4 construct fused to dihydrofolate reductase (DHFR) [96]. Since DHFR is a destabilizing domain, the AcrIIA4-DHFR fusion is rapidly degraded. TMP binding stabilizes the fusion protein, and AcrIIA4 becomes functional. Thus, the addition of TMP eventually blocks the SpCas9-mediated genome editing (Figure 6A). Importantly, this control method diminished the off-target DNA cleavage in cells. As AcrIIA4 disrupts the SpCas9−PAM interaction, this system was also applied to switch off the gene expression induced by dSpCas9-VPR [96]. Similarly, an FKBP-derived destabilizing domain (DD) was fused to AcrIIA4, and Shield-1 stabilized the DD-AcrIIA4 fusion protein to inhibit the gene activation by dSpCas9-VPR in a dose-dependent manner [97]. While these strategies efficiently switched off the dCas9-based transcriptional activation, the Cas9-based DNA cleavage could not be completely switched off, thus requiring further optimization of the system.

Song et al. reported Acr proteins fused with a ligand-dependent intein [98]. This fusion blocked the function of the Acr. When the cells are treated with 4-hydroxytamoxifen (4HT), the protein splicing is initiated and functional Acr is released (Figure 6B). This way, 4HT-induced inhibition of SpCas9, St3Cas9, and prime editor was demonstrated in cells, though background Acr activity was observed in the absence of 4HT [98].

As Cas9′s action is performed in the nucleus, Acr proteins are maximally active in the nucleus. Zhang et al. designed an AcrIIA4 fused to the hormone-binding domain of the human estrogen receptor (hER-HBD) [99]. This fusion is retained in the cytoplasm by the interaction of hER-HBD to cytoplasmic Hsp90. Upon binding of β-estradiol to hER-HBD, Hsp90 is displaced and the AcrIIA4-hER-HBD fusion is translocated to the nucleus where it blocks the dSpCas9-based gene expression in a dose-dependent manner in yeasts [99]. This proof-of-concept study has yet to be demonstrated in mammalian cells.

### 5.2. Conditional Deactivation of Acr Proteins by Small Molecules

On the other hand, small molecules can be used to deactivate Acr proteins and induce genome editing. Calvache et al. fused AcrIIA4 to minimal auxin-inducible degron (mAID), such that the fusion protein can be degraded by auxin treatment through the ubiquitin-proteasome pathway (Figure 6C) [100]. Indeed, treatment with auxin (indole-3-acetic acid) degraded the Acr fusion proteins and induced dCas9-based gene activation in plant cells. However, the narrow dynamic range of modulation requires further optimization of the system [100].

Although Acr protein-based systems are in the early stage of development, above examples demonstrate that Acr proteins are amenable to small-molecule control. We expect that many ingenious methods for Acr protein control would be reported in the near future to fully take advantage of Acr protein’s diversity and potency. However, it should also be considered that the extra Acr protein expression cassette needs to be delivered into cells, which may complicate the system optimization and in vivo applications.

## 6. Small-Molecule Enhancers of Precise Genome Editors

SpCas9 is the most widely used genome editor, owing to its robust activity in diverse cell types and less stringent PAM requirements. However, SpCas9 tends to display substantial off-target activities compared to other Cas nucleases [101]. Thus, Cas nucleases from other species, and dCas9- and nCas9-based genome editors that do not induce DSB (i.e., base editors and prime editors) have been extensively investigated for precise genome editing. Nevertheless, their activities are not as robust as SpCas9. Thus, small molecules that enhance their functions could be used for efficient and safe genome editing.

### 6.1. Small-Molecule Enhancers of Cas12a

Cas12a (formerly known as Cpf1) is intrinsically more specific than SpCas9 [102]. However, they are less active than SpCas9 with more stringent PAM requirements. Thus, methods that allow the use of Cas12a instead of Cas9 would allow more specific genome editing. Ma et al. performed a cell-based chemical screening to identify two small molecules (VE-822, an ATR kinase inhibitor, and AZD-7762, a CHEK1 kinase inhibitor) that enhance Cas12a-mediated precise genome editing up to sixfold in human pluripotent stem cells [103], although the effects of the kinase inhibitors on humans should be carefully investigated for in vivo therapeutic applications. Li et al. performed extensive molecular dynamics (MD) simulations to identify candidate enhancers of the Cas12a-mediated genome editing [104]. Experimental testing revealed that a compound, quinazoline2,4(1H,3H)-dione, enhanced the genome editing in cells, although its efficacy and potency are relatively low [104].

### 6.2. Small-Molecule Enhancers of Base Editors

Base editors induce base substitutions without introducing DSB, thus serving as a safe alternative to DSB- and HDR-mediated genome editing. However, the base-editing efficiency is low in many cases, delaying its practical applications. Zhao et al. performed a cell-based chemical screening to identify HDAC inhibitors (ricolinostat and nexturastat A) as enhancers of cytosine base editors [105]. The compounds induced robust base editing in diverse cell types, particularly in human primary T cells to correct a pathogenic mutation in *ABCA4* from Stargardt disease, and in mouse embryos to generate an albinism model [105]. Liu et al. also discovered HDAC inhibitors (nexturastat A and abexinostat) as enhancers of cytosine base editors and adenine base editors [106]. Interestingly, the compounds enhanced the product purity of a cytosine base editor (BE3) by suppressing the undesirable C-to-G conversion, further assuring safe genome editing [106]. Shin et al. used a fluorescent reporter-based cellular assay to screen a chemical library and identified several HDAC inhibitors including romidepsin, which increased the efficiency of adenine base editors and cytosine base editors [107]. Romidepsin also enhanced the product purity of a cytosine base editor, BE3 [107]. Intriguingly, all of these examples identified HDAC inhibitors as enhancers of base editors. However, the molecular mechanism of these observations is not clearly understood, though a study showed that HDAC inhibitors could increase the expression level of base editors [105]. The effect of these HDAC inhibitors on humans should be carefully investigated before their in vivo therapeutic applications.

### 6.3. Small-Molecule Enhancers of Prime Editors

While base editors can generate point mutations (A to G, C to T, or C to G), prime editors can introduce various types of sequence changes (base replacement, deletion, and insertion) without DSB. However, the lower activity of the prime editors hampers its widespread use, and small-molecule enhancers of the prime editors could be employed for safer genome editing. Liu et al. developed a cell-based assay to identify HDAC inhibitors (nexturastat A, vorinostat, abexinostat) that enhance the prime editing for deletions and insertions, but not for point mutations [106]. However, the enhancement was genomic loci-dependent due to the reasons not fully understood [106]. Because factors affecting the prime-editing efficiency are being revealed [108,109], other types of small-molecule enhancers of prime editors are expected in the near future.

## 7. Conclusions and Future Perspective

We have reviewed diverse small-molecule approaches used for precision genome editing (Table 1). Small molecules can control the expression level of genome editing machinery (i.e., Cas nucleases and gRNAs) at the transcription and translation stages. For more rapid regulation, Cas nucleases can be controlled after translation using small-molecule protein activators or inhibitors. Similarly, gRNAs can be controlled after transcription.

Owing to the cell-permeable properties of small molecules, many examples demonstrated swift, reversible, and dose-dependent control of genome editing. In some cases, small-molecule control could be combined with other tools to enable spatiotemporal control of genome editing, which is highly desirable for in vivo therapeutic applications.

In addition, combined small-molecule control both at the transcriptional level and the post-translational level was demonstrated for Cas9 proteins, such that background Cas9 activity was minimized to enable highly precise control. For example, the doxycycline-induced Cas9 expression system can be combined with DD-Cas9 fusion that is stabilized and activated by Shield-1 [64,110].

We also discussed emerging technologies that employ small molecule-responsive Acr proteins. To solve the problem of intracellular delivery, the engineered Acr constructs are delivered into cells along with Cas nucleases. The Acr proteins are conditionally activated by small molecules to enable temporal control of genome editing. Bacteriophages have evolved enormous numbers of Acr proteins with high efficacy and potency. Therefore, small molecule-controlled Acr proteins could find widespread use in precise genome editing.

Recently, precise genome editors that do not induce DSB are being extensively investigated. Indeed, an in vivo base editor therapeutics recently entered a clinical trial to correct the *PCSK9* gene from patients with heterozygous familial hypercholesterolaemia [111]. Prime editors have also been developed at an unprecedented speed since the first report in 2019 [112]. Small molecules that can solve the issues associated with these precise editors would greatly expand their utility. For example, molecules that reduce the gRNA-independent off-target nucleotide conversions and prevent the unproductive base exchanges could be discovered for enhancing base editors [113,114,115]. Molecules that direct the prime-editing repair pathway in a desirable direction could also be useful tools [116]. These small molecules could be rationally developed after careful investigation of the DNA repair mechanisms during the base editing and prime editing.

Several small-molecule modulators are approved drugs with well-known pharmacokinetic profiles and side effects (e.g., doxycycline, TMP) [117,118]. We expect that these molecules could be particularly useful for in vivo therapeutic applications. Other types of small molecules may be employed for ex vivo therapeutic editing (e.g., pluripotent stem-cell editing for cell replacement therapies, T-cell editing for cancer immunotherapies) and for cell-line editing to generate cellular model systems. Regardless of the intended applications, the effects of small molecules on cellular fitness should be carefully investigated, considering that small molecule display variable degree of side effects in different cells and organisms.

Currently, a majority of the switch-on systems do not display fully active genome editing even after the small-molecule induction, or exhibit leaky genome editing even without the small molecules. In the case of switch-off systems, the Cas nuclease activity could not be completely abolished, or the basal nuclease activity was diminished since the modified editing machineries were employed. In the future research, Cas nucleases or gRNAs containing minimal modification need to be developed for retaining their catalytic activities. In addition, careful titration of the editing machinery and small molecule is required for highly precise control of genome editing with minimal background activity.

## Figures and Tables

**Table 1:** Examples of small molecules that control the activity and duration of Cas nucleases and gRNAs/crRNAs.

**Small-molecule control of Cas9 expression levels**							
**Cas nuclease**	**RNA**	**Small molecule**	**Mode of action**		**Note**		**Refs.**
SpCas9, SaCas9, AsCas12a, RfxCas13d, base editor, prime editor	gRNA, crRNA, pegRNA	Doxycycline	Doxycycline-induced synthesis of mRNAs encoding the genome editors.		Extra expression of rtTA is required. Currently used in a wide assortment of biological systems in vitro and in vivo. Enhancement of the genome-editing specificity was demonstrated.		[25,26,27,28,29,30,31,32,33,34]
SpCas9	gRNA	Lys(Boc), an unnatural amino acid	Unnatural amino acid induces amber codon (UAG) suppression to generate full-length SpCas9.		Extra expression of aaRS and tRNA is required.		[35]
SpCas9, CjCas9	gRNA	G418	G418 induces stop codon (UGA) read-through to generate full-length Cas9.		Could affect other cellular pathways.		[36]
SpCas9, base editor, prime editor	gRNA, pegRNA	KPT330	KPT330 inhibits the export of mRNAs encoding the genome editors.		Could affect other cellular pathways. Enhancement of the genome-editing specificity was demonstrated.		[38]
**Small-molecule control of gRNA expression levels**							
**Cas nuclease**	**RNA**	**Small molecule**	**Mode of action**		**Note**		**Ref.**
SpCas9, SaCas9	gRNA	Doxycycline	Doxycycline-bound TetR dissociates from H1/TetO promoter, and gRNA transcription is initiated.		Extra expression of TetR is required.		[39,40,41]
SpCas9	gRNA	Doxycycline	Doxycycline-induced production of a gRNA targeting the SpCas9 gene.		Extra expression of TetR is required. Irreversible system; SpCas9 gene is disrupted. Enhancement of the genome-editing specificity was demonstrated.		[42]
SpCas9	gRNAs harboring LoxP sequences	4HT	Activation of Cre-ERT2 by 4HT leads to the recombination of gRNA-encoding genes, thus promoting or blocking the gRNA synthesis.		Extra expression of Cre-ERT2 is required. Enhancement of the genome-editing specificity was demonstrated.		[43]
**Small-molecule control of unmodified Cas nucleases**							
**Cas nuclease**	**RNA**	**Small molecule**	**Mode of action**		**Note**		**Ref.**
SpCas9, dSpCas9, base editor	gRNA	BRD0539, BRD20322, BRD7087	Inhibition of SpCas9−PAM interaction.		The first small-molecule SpCas9 inhibitor having potent cellular activities. Inhibitors of the various Cas9-based technologies. Sub-optimal efficacy and potency.		[21]
SpCas9	gRNA	Compound 85 and analogs	Inhibition of gRNA loading on SpCas9.		Inhibits SpCas9 in *E. coli*.		[45]
SpCas9	gRNA	6 compounds from UCLA Molecular Shared Screening Resource	Inhibition of SpCas9 with unknown mechanism.		Several hit compounds inhibit SpCas9 in test tubes, but their high toxicity restricted cellular tests.		[46]
SpCas9	gRNA	Valproic acid	Binds to SpCas9 to induce its thermal destabilization.		Photothermal triggers are required for efficient denaturation of SpCas9.		[47]
**Small-molecule control of engineered Cas nucleases**							
**Cas nuclease**	**RNA**	**Small molecule**	**Mode of action**		**Note**		**Ref.**
AID-dSpCas9, AID-dSpCas9-PR, AID-dSaCas9-VP64	gRNA	Auxin	Auxin-induced degradation of Cas9 proteins by the proteasome.		Rapid degradation of Cas9 proteins. Extra delivery of plant factors for ubiquitination is required.		[52]
SpCas9-FKBP^F36V^	gRNA	dTAG-47	dTAG-47-induced degradation of SpCas9-FKBPF36V by the proteasome.		Enhancement of the genome-editing specificity was demonstrated. DNA repair outcome was altered by modulating SpCas9′s half-life.		[55]
SpCas9-FCPF, dSpCas9-FCPF, PdCas12a-FCPF, LwCas13a-FCPF,	gRNA, crRNA	A conjugate of lenalidomide and perfluoroaromatic moiety	Conjugate-induced degradation of Cas proteins by the proteasome.		Applicable to diverse Cas nucleases.		[57]
SpCas9-DHFR, SpCas9-ER50	gRNA	TMP, 4HT	SpCas9-destabilizing domain fusions are stabilized by binding to small molecules.		Enhancement of the genome-editing specificity was demonstrated. Demonstrated in gene-drive applications.		[58,59]
SpCas9-DHFR	gRNA	Caged TMP molecules	SpCas9-DHFR fusion is stabilized by binding to TMP.		Spatiotemporal control was achieved using caged TMPs that release active TMP upon stimulation by reactive oxygen species or light. Demonstrated in vivo.		[60,61]
DD-SpCas9, DD-dSpCas9-VPR	gRNA	Shield-1	DD-Cas9 fusions are stabilized by binding to Shield-1.		Demonstrated in vivo.		[62,63,64]
SpCas9 fused to a 4HT-responsive intein	gRNA	4HT	4HT binding to the intein initiates the protein splicing to release active SpCas9.		Enhancement of the genome-editing specificity was demonstrated.		[65]
SpCas9, dSpCas9-VPR, LbCas12a, AsCas12a, dLbCas12a-p65-HSF1	gRNA, crRNA	Rapamycin	Rapamycin induces functional assembly of split Cas nucleases.		Demonstrated in vivo.		[67,68,69,70]
Base editors	gRNA	Rapamycin	Rapamycin induces functional assembly of split deaminases.		Genome-wide off-target base exchanges arising from the constitutively active deaminase were decreased.		[71,72]
SpCas9-ERT2, AsCas12a-ERT2	gRNA, crRNA	4HT	The binding of 4HT displaces the ERT2-bound cytoplasmic Hsp90 to localize the Cas-ERT2 fusions to the nucleus.		Enhancement of the genome-editing specificity was demonstrated.		[68,73,74]
BCL-xL and BH3 fused with SpCas9, dSpCas9-VPR, base editors, or prime editors	gRNA, pegRNA	A-385358, A1155463, WHEI-539	Autoinhibitory BCL-xL–BH3 interaction is inhibited by small molecules and Cas9 activity is restored.		Enhancement of the genome-editing specificity was demonstrated. Demonstrated with diverse dCas9 or nCas9-based technologies.		[75,76,77]
SpCas9 K866OABK mutant	gRNA	2DPBA, 2DPBM	2DPBA and 2DPBM are reacted with OABK to release functional lysine.		Extra expression of aaRS and tRNA is required when DNA is delivered. Appropriate for RNP delivery.		[79]
Base editor K1200TCOK mutant at nCas9 domain	gRNA	Me_2_Tz	Me_2_Tz is reacted with OABK to release functional lysine.		Extra expression of aaRS and tRNA is required when DNA is delivered. Appropriate for RNP delivery.		[80]
**Small-molecule control of engineered gRNAs**							
**Cas nuclease**	**RNA**	**Small molecule**	**Mode of action**		**Notes**		**Ref.**
SpCas9, dSpCas9	gRNA fused with an aptamer at the loop	Theophylline	Theophylline binding activates or deactivates gRNAs.		Demonstrated in *E. coli* for Cas9 nuclease activity and dCas9-based transcription modulation, and in human cells for dCas9-based transcription modulation.		[83,85,86]
SpCas9, dSpCas9-VPR	gRNA fused with an aptamer and a blocking motif at the 3′ end	Theophylline	Theophylline binding activates gRNAs.		Demonstrated in human cells for controlling the Cas9 nuclease activity.		[87]
SpCas9, dSpCas9-VPR, base editor	gRNA fused with an aptazyme at the 5′ end	Theophylline, guanine	Theophylline binding induces the self-cleavage by the aptazyme to release functional gRNAs.		Demonstrated in human cells for diverse Cas9-based technologies.		[88]
SpCas9	gRNA containing C-to-G mutations at stem-loops	Naphthyridine carbamate dimer (NCD)	NCD binding deactivates gRNAs and switches off genome editing.		Demonstrated in human cells for inhibiting Cas9 nuclease activity.		[89]
SpCas9, LbCas13a, dLbCas13a, LbCas12a	gRNA or crRNA chemically masked by AMN groups	2DPBM, TPPMS, THPP, TCEP	Phosphine compounds react with the AMN group to unmask and activate gRNAs/crRNAs		Demonstrated in human cells for various Cas proteins.		[90,91,92]
SpCas9, LbCas13a	Adamantoylated gRNA or crRNA	CB7	Host–guest complexation increased the bulkiness of gRNAs/crRNAs to inactivate them.		Demonstrated in human cells for inhibiting Cas9 nuclease activity.		[93]
SpCas9, LbCas13a	Azido-group-containing gRNA or crRNA	DBCO-containing molecule	Click chemistry increased the bulkiness of gRNAs/crRNAs to inactivate them.		Demonstrated in human cells for inhibiting Cas9 nuclease activity.		[94]
**Small-molecule control of anti-CRISPR proteins**							
**Cas nuclease**	**RNA**	**Acr protein**	**Small molecule**	**Mode of action**		**Notes**	**Ref.**
SpCas9	gRNA	AcrIIA4-DHFR	TMP	AcrIIA4-DHFR fusion is stabilized by TMP, and SpCas9 is inhibited.		Enhancement of the genome-editing specificity was demonstrated.	[96]
dSpCas9-VPR	gRNA	DD-AcrIIA4	Shield-1	DD-AcrIIA4 fusion is stabilized by Shield-1, and SpCas9 is inhibited.		Shield-1 dose-dependent inhibition of gene expression was demonstrated.	[97]
SpCas9, St3Cas9, prime editor	gRNA, pegRNA	AcrIIA25.1 or AcrIIA32.1 fused to a 4-HT-dependent intein	4HT	Acr proteins fused to a ligand-responsive intein are activated by binding to 4HT followed by protein splicing.		4HT-dependent inhibition of various Cas proteins was demonstrated.	[98]
dSpCas9	gRNA	AcrIIA4-hER-HBD	β-estradiol	AcrIIA4-hER-HBD is translocated to the nucleus by binding to β-estradiol.		Control of the dSpCas9-based gene expression was demonstrated in yeast.	[99]
dSpCas9	gRNA	AcrIIA4-mAID	auxin	AcrIIA4-mAID fusion is degraded by auxin, and cas9 becomes active.		Control of the dSpCas9-based gene expression was demonstrated in plant cells.	[100]
**Small-molecule enhancers of precise genome editors**							
**Cas nuclease**	**RNA**	**Small molecule**	**Mode of action**		**Notes**		**Ref.**
LbCas12a	crRNA	VE-822, AZD-7762	ATR kinase inhibitor, CHEK1 kinase inhibitor		Enhanced the editing efficiency up to 6-fold in human pluripotent stem cells. Not cytotoxic.		[103]
AsCas12a	crRNA	Quinazoline-2,4(1H,3H)-dione]	The compound stabilizes the Cas12a-crRNA complex.		Enhanced the editing efficiency up to ~1.8 fold in human cells.		[104]
Base editor	gRNA	Ricolinostat, nexturastat A	HDAC inhibitor		Induces robust base editing in diverse cell types, including human primary T cells and mouse embryos. Increased the expression level of base editors.		[105]
Base editor	gRNA	Nexturastat A, abexinostat	HDAC inhibitor		Enhanced the base-editing efficiency. Enhanced the product purity of BE3 by suppressing C-to-G conversion.		[106]
Base editor	gRNA	Romidepsin	HDAC inhibitor		Enhanced the base-editing efficiency. Enhanced the product purity of BE3.		[107]
Prime editor	pegRNA	Nexturastat A, vorinostat, abexinostat	HDAC inhibitor		Enhance the prime editing for deletions and insertions, but not for point mutations. Genomic loci-dependent enhancemnt.		[106]

## References

[1] Sharma G., Sharma A.R., Bhattacharya M., Lee S.-S., Chakraborty C. (2021). CRISPR-Cas9: A Preclinical and Clinical Perspective for the Treatment of Human Diseases. Mol. Ther..

[2] Modell A.E., Lim D., Nguyen T.M., Sreekanth V., Choudhary A. (2022). CRISPR-based therapeutics: Current challenges and future applications. Trends Pharmacol. Sci..

[3] Yeh C.D., Richardson C.D., Corn J.E. (2019). Advances in genome editing through control of DNA repair pathways. Nature.

[4] Yang H., Ren S., Yu S., Pan H., Li T., Ge S., Zhang J., Xia N. (2020). Methods Favoring Homology-Directed Repair Choice in Response to CRISPR/Cas9 Induced-Double Strand Breaks. Int. J. Mol. Sci..

[5] Lim D., Sreekanth V., Cox K.J., Law B.K., Wagner B.K., Karp J.M., Choudhary A. (2020). Engineering designer beta cells with a CRISPR-Cas9 conjugation platform. Nat. Commun..

[6] Anzalone A.V., Koblan L.W., Liu D.R. (2020). Genome editing with CRISPR-Cas nucleases, base editors, transposases and prime editors. Nat. Biotechnol..

[7] Liu G., Lin Q., Jin S., Gao C. (2022). The CRISPR-Cas toolbox and gene editing technologies. Mol. Cell.

[8] Herai R.H. (2019). Avoiding the off-target effects of CRISPR/cas9 system is still a challenging accomplishment for genetic trans-formation. Gene.

[9] Rayner E., Durin M.-A., Thomas R., Moralli D., O’Cathail S.M., Tomlinson I., Green C., Lewis A. (2019). CRISPR-Cas9 Causes Chromosomal Instability and Rearrangements in Cancer Cell Lines, Detectable by Cytogenetic Methods. CRISPR J..

[10] Charlesworth C.T., Deshpande P.S., Dever D.P., Camarena J., Lemgart V.T., Cromer M.K., Vakulskas C.A., Collingwood M.A., Zhang L., Bode N.M. (2019). Identification of preexisting adaptive immunity to Cas9 proteins in humans. Nat. Med..

[11] Wagner D.L., Amini L., Wendering D.J., Burkhardt L.-M., Akyüz L., Reinke P., Volk H.-D., Schmueck-Henneresse M. (2019). High prevalence of Streptococcus pyogenes Cas9-reactive T cells within the adult human population. Nat. Med..

[12] Tan R., Du W., Liu Y., Cong X., Bai M., Jiang C., Li Z., Tan M., Ma D.K., Huang Q. (2022). Nucleolus localization of SpyCas9 affects its stability and interferes with host protein translation in mammalian cells. Genes Dis..

[13] Kim N., Kim H.K., Lee S., Seo J.H., Choi J.W., Park J., Min S., Yoon S., Cho S.-R., Kim H.H. (2020). Prediction of the sequence-specific cleavage activity of Cas9 variants. Nat. Biotechnol..

[14] Schmid-Burgk J.L., Gao L., Li D., Gardner Z., Strecker J., Lash B., Zhang F. (2020). Highly Parallel Profiling of Cas9 Variant Specificity. Mol. Cell.

[15] Ryan D.E., Taussig D., Steinfeld I., Phadnis S.M., Lunstad B.D., Singh M., Vuong X., Okochi K.D., McCaffrey R., Olesiak M. (2018). Improving CRISPR-Cas specificity with chemical modifications in single-guide RNAs. Nucleic Acids Res..

[16] Cromwell C.R., Sung K., Park J., Krysler A.R., Jovel J., Kim S.K., Hubbard B.P. (2018). Incorporation of bridged nucleic acids into CRISPR RNAs improves Cas9 endonuclease specificity. Nat. Commun..

[17] Kocak D.D., Josephs E.A., Bhandarkar V., Adkar S.S., Kwon J.B., Gersbach C.A. (2019). Increasing the specificity of CRISPR systems with engineered RNA secondary structures. Nat. Biotechnol..

[18] Shin J., Jiang F., Liu J.-J., Bray N.L., Rauch B.J., Baik S.H., Nogales E., Bondy-Denomy J., Corn J.E., Doudna J.A. (2017). Disabling Cas9 by an anti-CRISPR DNA mimic. Sci. Adv..

[19] Aschenbrenner S., Kallenberger S.M., Hoffmann M.D., Huck A., Eils R., Niopek D. (2020). Coupling Cas9 to artificial inhibitory domains enhances CRISPR-Cas9 target specificity. Sci. Adv..

[20] Marino N., Pinilla-Redondo R., Csörgő B., Bondy-Denomy J. (2020). Anti-CRISPR protein applications: Natural brakes for CRISPR-Cas technologies. Nat. Methods.

[21] Maji B., Gangopadhyay S.A., Lee M., Shi M., Wu P., Heler R., Mok B., Lim D., Siriwardena S.U., Paul B. (2019). A High-Throughput Platform to Identify Small-Molecule Inhibitors of CRISPR-Cas9. Cell.

[22] Jaeger M.G., Winter G.E. (2021). Fast-acting chemical tools to delineate causality in transcriptional control. Mol. Cell.

[23] Gangopadhyay S.A., Cox K.J., Manna D., Lim D., Maji B., Zhou Q., Choudhary A. (2019). Precision Control of CRISPR-Cas9 Using Small Molecules and Light. Biochemistry.

[24] Modell A.E., Siriwardena S.U., Shoba V.M., Li X., Choudhary A. (2021). Chemical and optical control of CRISPR-associated nucleases. Curr. Opin. Chem. Biol..

[25] Dow L.E., Fisher J., O’Rourke K.P., Muley A., Kastenhuber E.R., Livshits G., Tschaharganeh D.F., Socci N.D., Lowe S.W. (2015). Inducible in vivo genome editing with CRISPR-Cas9. Nat. Biotechnol..

[26] Cao J., Wu L., Zhang S.-M., Lu M., Cheung W.K., Cai W., Gale M., Xu Q., Yan Q. (2016). An easy and efficient inducible CRISPR/Cas9 platform with improved specificity for multiple gene targeting. Nucleic Acids Res..

[27] Hundley F.V., Delgado N.S., Marin H.C., Carr K.L., Tian R., Toczyski D.P. (2021). A comprehensive phenotypic CRISPR-Cas9 screen of the ubiquitin pathway uncovers roles of ubiquitin ligases in mitosis. Mol. Cell.

[28] Zhou Z., Li Y., Xu H., Xie X., He Z., Lin S., Li R., Jin S., Cui J., Hu H. (2022). An inducible CRISPR/Cas9 screen identifies DTX2 as a transcriptional regulator of human telomerase. iScience.

[29] Lundin A., Porritt M.J., Jaiswal H., Seeliger F., Johansson C., Bidar A.W., Badertscher L., Wimberger S., Davies E.J., Hardaker E. (2020). Development of an ObLiGaRe Doxycycline Inducible Cas9 system for pre-clinical cancer drug discovery. Nat. Commun..

[30] Li Y.S., Meng R.R., Chen X., Shang C.L., Li H.B., Zhang T.J., Long H.Y., Li H.Q., Wang Y.J., Wang F.C. (2019). Generation of H11-albumin-rtTA Transgenic Mice: A Tool for Inducible Gene Expression in the Liver. G3 Genes Genomes Genet..

[31] Sun H., Fu S., Cui S., Yin X., Sun X., Qi X., Cui K., Wang J., Ma L., Liu F.Y. (2020). Development of a CRISPR-SaCas9 system for projection- and function-specific gene editing in the rat brain. Sci. Adv..

[32] Hughes N.W., Qu Y., Zhang J., Tang W., Pierce J., Wang C., Agrawal A., Morri M., Neff N., Winslow M.M. (2022). Machine-learning-optimized Cas12a barcoding enables the recovery of single-cell lineages and tran-scriptional profiles. Mol. Cell.

[33] Zafra M.P., Schatoff E.M., Katti A., Foronda M., Breinig M., Schweitzer A.Y., Simon A., Han T., Goswami S., Montgomery E. (2018). Optimized base editors enable efficient editing in cells, organoids and mice. Nat. Biotechnol..

[34] Habib O., Habib G., Hwang G.-H., Bae S. (2022). Comprehensive analysis of prime editing outcomes in human embryonic stem cells. Nucleic Acids Res..

[35] Suzuki T., Asami M., Patel S.G., Luk L.Y.P., Tsai Y.-H., Perry A.C.F. (2018). Switchable genome editing via genetic code expansion. Sci. Rep..

[36] Yaméogo P., Duchêne B.L., Majeau N., Tremblay J.P. (2022). CRISPR-SCReT (CRISPR-Stop Codon Read Through) method to control Cas9 expression for gene editing. Gene Ther..

[37] Friesen W.J., Johnson B., Sierra J., Zhuo J., Vazirani P., Xue X., Tomizawa Y., Baiazitov R., Morrill C., Ren H. (2018). The minor gentamicin complex component, X2, is a potent premature stop codon readthrough molecule with therapeutic potential. PLoS ONE.

[38] Cui Y.-R., Wang S.-J., Ma T., Yu P., Chen J., Guo T., Meng G., Jiang B., Dong J., Liu J. (2022). KPT330 improves Cas9 precision genome- and base-editing by selectively regulating mRNA nuclear export. Commun. Biol..

[39] De Solis C.A., Ho A., Holehonnur R., Ploski J.E. (2016). The Development of a Viral Mediated CRISPR/Cas9 System with Doxycycline Dependent gRNA Expression for Inducible In vitro and In vivo Genome Editing. Front. Mol. Neurosci..

[40] Aubrey B.J., Kelly G.L., Kueh A.J., Brennan M.S., O’Connor L., Milla L., Wilcox S., Tai L., Strasser A., Herold M.J. (2015). An inducible lentiviral guide RNA platform enables the identification of tumor-essential genes and tu-mor-promoting mutations in vivo. Cell Rep..

[41] Kumar N., Stanford W., De Solis C., Aradhana Abraham N.D., Dao T.-M.J., Thaseen S., Sairavi A., Gonzalez C.U., Ploski J.E. (2018). The Development of an AAV-Based CRISPR SaCas9 Genome Editing System That Can Be Delivered to Neurons in vivo and Regulated via Doxycycline and Cre-Recombinase. Front. Mol. Neurosci..

[42] Kelkar A., Zhu Y., Groth T., Stolfa G., Stablewski A.B., Singhi N., Nemeth M., Neelamegham S. (2020). Doxycycline-Dependent Self-Inactivation of CRISPR-Cas9 to Temporally Regulate On- and Off-Target Editing. Mol. Ther..

[43] Chylinski K., Hubmann M., Hanna R., Yanchus C., Michlits G., Uijttewaal E.C.H., Doench J., Schramek D., Elling U. (2019). CRISPR-Switch regulates sgRNA activity by Cre recombination for sequential editing of two loci. Nat. Commun..

[44] Jia N., Patel D.J. (2021). Structure-based functional mechanisms and biotechnology applications of anti-CRISPR proteins. Nat. Rev. Mol. Cell Biol..

[45] Lee S.-W., Tran K.T., Vazquez-Uribe R., Gotfredsen C.H., Clausen M.H., Mendez B.L., Montoya G., Bach A., Sommer M.O.A. (2022). Identification and Optimization of Novel Small-Molecule Cas9 Inhibitors by Cell-Based High-Throughput Screening. J. Med. Chem..

[46] Seamon K.J., Light Y.K., Saada E.A., Schoeniger J.S., Harmon B. (2018). Versatile High-Throughput Fluorescence Assay for Monitoring Cas9 Activity. Anal. Chem..

[47] Cheng X. (2020). Valproic Acid Thermally Destabilizes and Inhibits SpyCas9 Activity. Mol. Ther..

[48] Vyas P. (2022). Anti-CRISPR proteins as a therapeutic agent against drug-resistant bacteria. Microbiol. Res..

[49] Qin S., Liu Y., Chen Y., Hu J., Xiao W., Tang X., Li G., Lin P., Pu Q., Wu Q. (2022). Engineered Bacteriophages Containing Anti-CRISPR Suppress Infection of Antibiotic-Resistant *P. aeruginosa*. Microbiol. Spectr..

[50] Cox K.J., Subramanian H.K.K., Samaniego C.C., Franco E., Choudhary A. (2019). A universal method for sensitive and cell-free detection of CRISPR-associated nucleases. Chem. Sci..

[51] Békés M., Langley D.R., Crews C.M. (2022). PROTAC targeted protein degraders: The past is prologue. Nat. Rev. Drug Discov..

[52] Kleinjan D.A., Wardrope C., Sou S.N., Rosser S.J. (2017). Drug-tunable multidimensional synthetic gene control using inducible degron-tagged dCas9 effectors. Nat. Commun..

[53] Nabet B., Ferguson F.M., Seong B.K.A., Kuljanin M., Leggett A.L., Mohardt M.L., Robichaud A., Conway A.S., Buckley D.L., Mancias J.D. (2020). Rapid and direct control of target protein levels with VHL-recruiting dTAG molecules. Nat. Commun..

[54] Nabet B., Roberts J.M., Buckley D.L., Paulk J., Dastjerdi S., Yang A., Leggett A.L., Erb M.A., Lawlor M., Souza A. (2018). The dTAG system for immediate and target-specific protein degradation. Nat. Chem. Biol..

[55] Sreekanth V., Zhou Q., Kokkonda P., Bermudez-Cabrera H.C., Lim D., Law B.K., Holmes B.R., Chaudhary S.K., Pergu R., Leger B.S. (2020). Chemogenetic System Demonstrates That Cas9 Longevity Impacts Genome Editing Outcomes. ACS Cent. Sci..

[56] Zhang S., Shen J., Li D., Cheng Y. (2021). Strategies in the delivery of Cas9 ribonucleoprotein for CRISPR/Cas9 genome editing. Theranostics.

[57] Gama-Brambila R.A., Chen J., Dabiri Y., Tascher G., Němec V., Münch C., Song G., Knapp S., Cheng X. (2021). A Chemical Toolbox for Labeling and Degrading Engineered Cas Proteins. JACS Au.

[58] Maji B., Moore C.L., Zetsche B., Volz S.E., Zhang F., Shoulders M.D., Choudhary A. (2017). Multidimensional chemical control of CRISPR–Cas9. Nat. Chem. Biol..

[59] Del Amo V.L., Leger B.S., Cox K.J., Gill S., Bishop A.L., Scanlon G.D., Walker J.A., Gantz V.M., Choudhary A. (2020). Small-Molecule Control of Super-Mendelian Inheritance in Gene Drives. Cell Rep..

[60] Yan X., Pan Q., Xin H., Chen Y., Ping Y. (2021). Genome-editing prodrug: Targeted delivery and conditional stabilization of CRISPR-Cas9 for precision therapy of inflammatory disease. Sci. Adv..

[61] Manna D., Maji B., Gangopadhyay S.A., Cox K.J., Zhou Q., Law B.K., Mazitschek R., Choudhary A. (2019). A Singular System with Precise Dosing and Spatiotemporal Control of CRISPR-Cas9. Angew. Chem. Int. Ed..

[62] Senturk S., Shirole N.H., Nowak D.G., Corbo V., Pal D., Vaughan A., Tuveson D.A., Trotman L.C., Kinney J.B., Sordella R. (2017). Rapid and tunable method to temporally control gene editing based on conditional Cas9 stabilization. Nat. Commun..

[63] Siolas D., Vucic E., Kurz E., Hajdu C., Bar-Sagi D. (2021). Gain-of-function p53R172H mutation drives accumulation of neutrophils in pancreatic tumors, promoting resistance to immunotherapy. Cell Rep..

[64] Holoch D., Wassef M., Lövkvist C., Zielinski D., Aflaki S., Lombard B., Héry T., Loew D., Howard M., Margueron R. (2021). A cis-acting mechanism mediates transcriptional memory at Polycomb target genes in mammals. Nat. Genet..

[65] Davis K.M., Pattanayak V., Thompson D.B., Zuris J.A., Liu D.R. (2015). Small molecule–triggered Cas9 protein with improved genome-editing specificity. Nat. Chem. Biol..

[66] Asp M.L., Martindale J.J., Metzger J.M. (2013). Direct, Differential Effects of Tamoxifen, 4-Hydroxytamoxifen, and Raloxifene on Cardiac Myocyte Contractility and Calcium Handling. PLoS ONE.

[67] Zetsche B., Volz S.E., Zhang F. (2015). A split-Cas9 architecture for inducible genome editing and transcription modulation. Nat. Biotechnol..

[68] Nguyen D.P., Miyaoka Y., Gilbert L., Mayerl S.J., Lee B.H., Weissman J.S., Conklin B.R., Wells J.A. (2016). Ligand-binding domains of nuclear receptors facilitate tight control of split CRISPR activity. Nat. Commun..

[69] Huynh N., Wang S., King-Jones K. (2020). Spatial and temporal control of gene manipulation in Drosophila via drug-activated Cas9 nucleases. Insect Biochem. Mol. Biol..

[70] Nihongaki Y., Otabe T., Ueda Y., Sato M. (2019). A split CRISPR–Cpf1 platform for inducible genome editing and gene activation. Nat. Chem. Biol..

[71] Berríos K.N., Evitt N.H., DeWeerd R.A., Ren D., Luo M., Barka A., Wang T., Bartman C.R., Lan Y., Green A.M. (2021). Controllable genome editing with split-engineered base editors. Nat. Chem. Biol..

[72] Long J., Liu N., Tang W., Xie L., Qin F., Zhou L., Tao R., Wang Y., Hu Y., Jiao Y. (2021). A split cytosine deaminase architecture enables robust inducible base editing. FASEB J..

[73] Liu K.I., Bin Ramli M.N., Woo C.W.A., Wang Y., Zhao T., Zhang X., Yim G.R.D., Chong B.Y., Gowher A., Chua M.Z.H. (2016). A chemical-inducible CRISPR–Cas9 system for rapid control of genome editing. Nat. Chem. Biol..

[74] Dominguez-Monedero A., Davies J.A. (2018). Tamoxifen- and Mifepristone-Inducible Versions of CRISPR Effectors, Cas9 and Cpf1. ACS Synth. Biol..

[75] Rose J.C., Stephany J.J., Valente W.J., Trevillian B.M., Dang H.V., Bielas J.H., Maly D.J., Fowler D.M. (2017). Rapidly inducible Cas9 and DSB-ddPCR to probe editing kinetics. Nat. Methods.

[76] Rose J.C., Stephany J.J., Wei C.T., Fowler D.M., Maly D.J. (2018). Rheostatic Control of Cas9-Mediated DNA Double Strand Break (DSB) Generation and Genome Editing. ACS Chem. Biol..

[77] Wei C.T., Peleg O., Borenstein E., Maly D.J., Fowler D.M. (2022). A versatile, chemically-controlled DNA binding switch enables temporal modulation of Cas9-based effectors. bioRxiv.

[78] Senichkin V.V., Pervushin N.V., Zuev A.P., Zhivotovsky B., Kopeina G.S. (2020). Targeting Bcl-2 Family Proteins: What, Where, When?. Biochemistry.

[79] Luo J., Liu Q., Morihiro K., Deiters J.L.Q.L.K.M.A. (2016). Small-molecule control of protein function through Staudinger reduction. Nat. Chem..

[80] Ngai W.S.C., Yang S., Zeng X., Liu Y., Lin F., Wang X., Zhang H., Fan X., Chen P.R. (2022). Bioorthogonally Activatable Base Editing for On-Demand Pyroptosis. J. Am. Chem. Soc..

[81] Trapani I. (2019). Adeno-Associated Viral Vectors as a Tool for Large Gene Delivery to the Retina. Genes.

[82] Schmidt M.J., Gupta A., Bednarski C., Gehrig-Giannini S., Richter F., Pitzler C., Gamalinda M., Galonska C., Takeuchi R., Wang K. (2021). Improved CRISPR genome editing using small highly active and specific engineered RNA-guided nucle-ases. Nat. Commun..

[83] Kundert K., Lucas J.E., Watters K.E., Fellmann C., Ng A.H., Heineike B.M., Fitzsimmons C.M., Oakes B.L., Qu J., Prasad N. (2019). Controlling CRISPR-Cas9 with ligand-activated and ligand-deactivated sgRNAs. Nat. Commun..

[84] Konermann S., Brigham M., Trevino A.E., Joung J., Abudayyeh O.O., Barcena C., Hsu P., Habib N., Gootenberg J., Nishimasu H. (2015). Genome-scale transcriptional activation by an engineered CRISPR-Cas9 complex. Nature.

[85] Iwasaki R.S., Ozdilek B.A., Garst A.D., Choudhury A., Batey R.T. (2020). Small molecule regulated sgRNAs enable control of genome editing in E. coli by Cas9. Nat. Commun..

[86] Liu Y., Wang Y., Lin J., Xu L. (2021). Theophylline-induced synergic activation of guide RNA to control CRISPR/Cas9 function. Chem. Commun..

[87] Lin B., An Y., Meng L., Zhang H., Song J., Zhu Z., Liu W., Song Y., Yang C. (2019). Control of CRISPR-Cas9 with small molecule-activated allosteric aptamer regulating sgRNAs. Chem. Commun..

[88] Tang W., Hu J.H., Liu D.R. (2017). Aptazyme-embedded guide RNAs enable ligand-responsive genome editing and transcriptional activation. Nat. Commun..

[89] Liu X., Xiong W., Qi Q., Zhang Y., Ji H., Cui S., An J., Sun X., Yin H., Tian T. (2022). Rational guide RNA engineering for small-molecule control of CRISPR/Cas9 and gene editing. Nucleic Acids Res..

[90] Wang S.-R., Wu L.-Y., Huang H.-Y., Xiong W., Liu J., Wei L., Yin P., Tian T., Zhou X. (2020). Conditional control of RNA-guided nucleic acid cleavage and gene editing. Nat. Commun..

[91] Habibian M., McKinlay C., Blake T.R., Kietrys A.M., Waymouth R.M., Wender P.A., Kool E.T. (2019). Reversible RNA acylation for control of CRISPR–Cas9 gene editing. Chem. Sci..

[92] Xie S., Xu B., Tang R., Chen S., Lei C., Nie Z. (2022). Kinetics Accelerated CRISPR-Cas12a Enabling Live-Cell Monitoring of Mn^2+^ Homeostasis. Anal. Chem..

[93] Xiong W., Liu X., Qi Q., Ji H., Liu F., Zhong C., Liu S., Tian T., Zhou X. (2022). Supramolecular CRISPR-OFF switches with host–guest chemistry. Nucleic Acids Res..

[94] Liu X.-Y., Xiong W., Qi Q.-Q., Ji H.-M., Zhang Y.-T., Lei H.-J., Liu J., Yin P., Tian T., Zhou X. (2022). A chemical CRISPR off switch efficiently controls gene editing. Cell Rep. Phys. Sci..

[95] Yang H., Patel D.J. (2017). Inhibition Mechanism of an Anti-CRISPR Suppressor AcrIIA4 Targeting SpyCas9. Mol. Cell.

[96] Jain S., Xun G., Abesteh S., Ho S., Lingamaneni M., Martin T.A., Tasan I., Yang C., Zhao H. (2021). Precise Regulation of Cas9-Mediated Genome Engineering by Anti-CRISPR-Based Inducible CRISPR Controllers. ACS Synth. Biol..

[97] Nakamura M., Srinivasan P., Chavez M., Carter M., Dominguez A.A., La Russa M., Lau M.B., Abbott T.R., Xu X., Zhao D. (2019). Anti-CRISPR-mediated control of gene editing and synthetic circuits in eukaryotic cells. Nat. Commun..

[98] Song G., Zhang F., Tian C., Gao X., Zhu X., Fan D., Tian Y. (2022). Discovery of potent and versatile CRISPR–Cas9 inhibitors engineered for chemically controllable genome editing. Nucleic Acids Res..

[99] Zhang Y., Marchisio M.A. (2022). Interaction of Bare dSpCas9, Scaffold gRNA, and Type II Anti-CRISPR Proteins Highly Favors the Control of Gene Expression in the Yeast S. cerevisiae. ACS Synth. Biol..

[100] Calvache C., Vazquez-Vilar M., Selma S., Uranga M., Fernández-Del-Carmen A., Daròs J., Orzáez D. (2021). Strong and tunable anti-CRISPR/Cas activities in plants. Plant Biotechnol. J..

[101] Vicente M.M., Chaves-Ferreira M., Jorge J.M.P., Proença J.T., Barreto V.M. (2021). The Off-Targets of Clustered Regularly In-terspaced Short Palindromic Repeats Gene Editing. Front. Cell Dev. Biol..

[102] Safari F., Zare K., Negahdaripour M., Barekati-Mowahed M., Ghasemi Y. (2019). CRISPR Cpf1 proteins: Structure, function and implications for genome editing. Cell Biosci..

[103] Ma X., Chen X., Jin Y., Ge W., Wang W., Kong L., Ji J., Guo X., Huang J., Feng X.-H. (2018). Small molecules promote CRISPR-Cpf1-mediated genome editing in human pluripotent stem cells. Nat. Commun..

[104] Li W., Chan C., Zeng C., Turk R., Behlke M.A., Cheng X., Dong Y. (2020). Rational Design of Small Molecules to Enhance Genome Editing Efficiency by Selectively Targeting Distinct Functional States of CRISPR-Cas12a. Bioconjugate Chem..

[105] Zhao T., Li Q., Zhou C., Lv X., Liu H., Tu T., Tang N., Cheng Y., Liu X., Liu C. (2021). Small-molecule compounds boost genome-editing efficiency of cytosine base editor. Nucleic Acids Res..

[106] Liu N., Zhou L., Lin G., Hu Y., Jiao Y., Wang Y., Liu J., Yang S., Yao S. (2022). HDAC inhibitors improve CRISPR-Cas9 mediated prime editing and base editing. Mol. Ther. Nucleic Acids.

[107] Shin H.R., See J.-E., Kweon J., Kim H.S., Sung G.-J., Park S., Jang A.-H., Jang G., Choi K.-C., Kim I. (2021). Small-molecule inhibitors of histone deacetylase improve CRISPR-based adenine base editing. Nucleic Acids Res..

[108] Da Silva J.F., Oliveira G.P., Arasa-Verge E.A., Kagiou C., Moretton A., Timelthaler G., Jiricny J., Loizou J.I. (2022). Prime editing efficiency and fidelity are enhanced in the absence of mismatch repair. Nat. Commun..

[109] Chen P.J., Hussmann J.A., Yan J., Knipping F., Ravisankar P., Chen P.F., Chen C., Nelson J.W., Newby G.A., Sahin M. (2021). Enhanced prime editing systems by manipulating cellular determinants of editing outcomes. Cell.

[110] Park S.B., Uchida T., Tilson S., Hu Z., Ma C.D., Leek M., Eichner M., Hong S.G., Liang T.J. (2022). A dual conditional CRISPR-Cas9 system to activate gene editing and reduce off-target effects in human stem cells. Mol. Ther. Nucleic Acids.

[111] Kingwell K. (2022). Base editors hit the clinic. Nat. Rev. Drug Discov..

[112] Anzalone A.V., Randolph P.B., Davis J.R., Sousa A.A., Koblan L.W., Levy J.M., Chen P.J., Wilson C., Newby G.A., Raguram A. (2019). Search-and-replace genome editing without double-strand breaks or donor DNA. Nature.

[113] Alvarez-Gonzalez J., Yasgar A., Maul R.W., Rieffer A.E., Crawford D.J., Salamango D.J., Dorjsuren D., Zakharov A.V., Jansen D.J., Rai G. (2021). Small Molecule Inhibitors of Activation-Induced Deaminase Decrease Class Switch Recombination in B Cells. ACS Pharmacol. Transl. Sci..

[114] Wang L., Xue W., Zhang H., Gao R., Qiu H., Wei J., Zhou L., Lei Y.-N., Wu X., Li X. (2021). Eliminating base-editor-induced genome-wide and transcriptome-wide off-target mutations. Nat..

[115] Li A., Mitsunobu H., Yoshioka S., Suzuki T., Kondo A., Nishida K. (2022). Cytosine base editing systems with minimized off-target effect and molecular size. Nat. Commun..

[116] Peterka M., Akrap N., Li S., Wimberger S., Hsieh P.-P., Degtev D., Bestas B., Barr J., van de Plassche S., Mendoza-Garcia P. (2022). Harnessing DSB repair to promote efficient homology-dependent and -independent prime editing. Nat. Commun..

[117] Henehan M., Montuno M., De Benedetto A. (2017). Doxycycline as an anti-inflammatory agent: Updates in dermatology. J. Eur. Acad. Dermatol. Venereol..

[118] Smilack J.D. (1999). Trimethoprim-sulfamethoxazole. Mayo. Clin. Proc..

